# Inter-Vendor Reproducibility of Pseudo-Continuous Arterial Spin Labeling at 3 Tesla

**DOI:** 10.1371/journal.pone.0104108

**Published:** 2014-08-04

**Authors:** Henri J. M. M. Mutsaerts, Rebecca M. E. Steketee, Dennis F. R. Heijtel, Joost P. A. Kuijer, Matthias J. P. van Osch, Charles B. L. M. Majoie, Marion Smits, Aart J. Nederveen

**Affiliations:** 1 Department of Radiology, Academic Medical Center, Amsterdam, The Netherlands; 2 Department of Radiology, Erasmus MC University Medical Center Rotterdam, Rotterdam, The Netherlands; 3 Department of Physics and Medical Technology, VU University Medical Center, Amsterdam, The Netherlands; 4 C. J. Gorter Center for High Field MRI, Department of Radiology, Leiden University Medical Center, Leiden, The Netherlands; University of Texas at Dallas, United States of America

## Abstract

**Purpose:**

Prior to the implementation of arterial spin labeling (ASL) in clinical multi-center studies, it is important to establish its status quo inter-vendor reproducibility. This study evaluates and compares the intra- and inter-vendor reproducibility of pseudo-continuous ASL (pCASL) as clinically implemented by GE and Philips.

**Material and Methods:**

22 healthy volunteers were scanned twice on both a 3T GE and a 3T Philips scanner. The main difference in implementation between the vendors was the readout module: spiral 3D fast spin echo vs. 2D gradient-echo echo-planar imaging respectively. Mean and variation of cerebral blood flow (CBF) were compared for the total gray matter (GM) and white matter (WM), and on a voxel-level.

**Results:**

Whereas the mean GM CBF of both vendors was almost equal (*p* = 1.0), the mean WM CBF was significantly different (*p*<0.01). The inter-vendor GM variation did not differ from the intra-vendor GM variation (*p* = 0.3 and *p* = 0.5 for GE and Philips respectively). Spatial inter-vendor CBF and variation differences were observed in several GM regions and in the WM.

**Conclusion:**

These results show that total GM CBF-values can be exchanged between vendors. For the inter-vendor comparison of GM regions or WM, these results encourage further standardization of ASL implementation among vendors.

## Introduction

Arterial spin labeling (ASL) is an emerging magnetic resonance imaging (MRI) perfusion modality that enables non-invasive cerebral perfusion measurements. Since ASL is virtually harmless, not hampered by the blood-brain barrier and enables absolute quantification of cerebral blood flow (CBF), it is an attractive tool compared to other perfusion imaging modalities [1], [2]. Through several methodological advances, ASL perfusion MRI has matured to the point where it can provide high quality whole-brain perfusion images in only a few minutes of scanning [3]. Its reproducibility has been established and its CBF-maps are comparable with imaging methods based on exogenous tracers [4]–[7]. ASL is commercially available on all major MRI systems and clinical applications are under rapid development. ASL-based CBF measurements are of clinical value in a number of cerebral pathologies, such as brain tumors, cerebrovascular pathology, epilepsy and neurodegeneration [8], [9]. Therefore, the initiation of large-scale multi-center ASL studies is a next step to extend our understanding of the pathophysiology of many common disorders.

However, it is essential to first establish the inter-vendor reproducibility of ASL [10], [11]. One main obstacle that impedes multi-center studies, is that fundamental differences exist between ASL implementations of different vendors. Each MRI vendor has implemented a different labeling-readout combination, which may seriously hamper the comparison of multi-vendor ASL-data [12]. Since each labeling and readout strategy exhibits specific advantages and disadvantages, a substantial technical heterogeneity is introduced [13]. Therefore, it remains unclear to which degree ASL-based CBF-maps from centers with scanners of different vendors are comparable. The aim of the current study is to assess and compare the intra- and inter-vendor reproducibility of pseudo-continuous ASL (pCASL) CBF measurements as currently clinically implemented by two major vendors: i.e. GE and Philips.

## Materials and Methods

### Subject recruitment and study design

Twenty-two healthy volunteers (9 men, 13 women, mean age 22.6±2.1 (SD) years) were included. In addition to standard MRI exclusion criteria, subjects with history of brain or psychiatric disease or use of medication - except for oral contraceptives - were excluded. No consumption of vasomotor substances such as alcohol, cigarettes, coffee, licorice and tea was allowed on the scan days. On the day prior to the examination, alcohol and nicotine consumption was restricted to three units and cigarettes respectively.

All subjects were scanned twice at two academic medical centers in the Netherlands: Erasmus MC – University Medical Center Rotterdam (center 1) and Academic Medical Center Amsterdam (center 2). The inter-session time interval was kept at 1–4 weeks. MRI experiments were performed on a 3T GE scanner at center 1 (Discovery MR750, GE Healthcare, Milwaukee, WI, US) and on a 3T Philips scanner at center 2 (Intera, Philips Healthcare, Best, the Netherlands), both equipped with an 8-channel head coil (InVivo, Gainesville, FL, US). Foam padding inside the head coil was used to restrict head motion during scanning [10]. Subjects were awake and had their eyes closed during all ASL scans.

### Ethics statement

All subjects provided written informed consent and the study was approved by the ethical review boards of both centers.

### Acquisition

**Figure 1:**
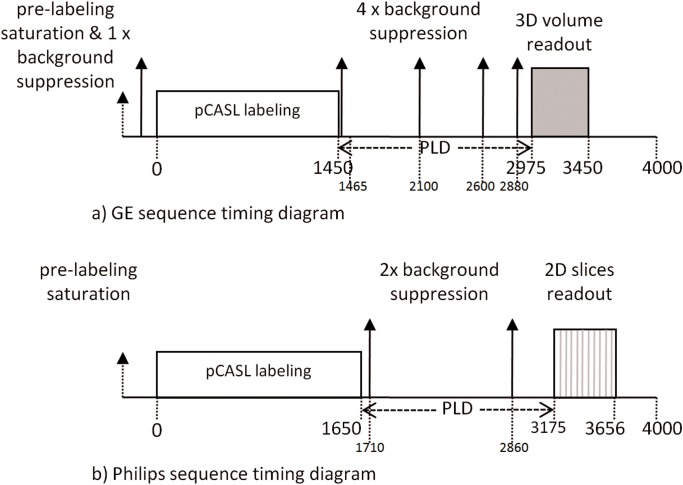
Sequence timing diagrams of a) General Electric (GE) and b) Philips, shown at the same time scale (ms). pCASL = pseudo-continuous arterial spin labeling, PLD = post-labeling delay.

Each scan session included a pCASL and 1 mm isotropic 3D T1-weighted scan for segmentation and registration purposes. For the acquisition of a single time-point CBF-map, pCASL has become the preferred labeling strategy because of its relatively high signal-to-noise ratio (SNR) and wide availability across all platforms [3], [14]. On both scanners we employed the clinically implemented pCASL protocols that are currently used in clinical studies [15], [16]. Table 1 and Figure 1 summarize the protocol details and show the timing diagrams for both sequences respectively. The main difference between the GE and Philips implementations was the readout module: multi-shot spiral 3D fast spin-echo vs. single-shot 2D gradient-echo echo-planar imaging respectively.

**Table 1:** Acquisition protocols.

	GE	Philips
Labeling module	pseudo-continuous	pseudo-continuous
Labeling pulse shape	Hanning	Hanning
Labeling pulse duration	0.5 ms	0.5 ms
Labeling pulse flip angle	23°	18°
Mean gradient strength	0.7 mT/m	0.6 mT/m
Maximal gradient strength	7 mT/m	6 mT/m
Labeling duration	1450 ms	1650 ms
Post-labeling delay (PLD) (initial)	1525 ms	1525 ms
PLD increase per slice	n.a.	28.3 ms
PLD (average)	1525 ms	1770 ms
Labeling plane planning	Fixed 22 mm below lower edge	89 mm below, parallel to AC-PC line
Labeling plane distance*	72 mm	89 mm
Readout module	3D fast spin-echo interleaved stack-of-spirals	2D gradient-echo single-shot echo-planar imaging
		SENSE 2.5, CLEAR
Acquisition matrix	8 spirals×512 sampling points	80×80
Field of view	24 cm^3^	24 cm^2^
Number of slices	36	17
Slice thickness	4 mm	7 mm
Acquisition voxel size (volume)	3.8×3.8×4 mm (57.8 mm^3^)	3.0×3.0×7.0 mm (63 mm^3^)
Reconstruction voxel size	1.9×1.9×4.0 mm	3.0×3.0×7.0 mm
Slice gap	n.a.	0 mm
Echo time/repetition time	10.5/4600 ms	17/4000 ms
Number of signal averages	3	33
Readout planning	True axial, lower edge fixed at lower boundary pons	Parallel to AC-PC line
Background suppression (n pulses)	yes (5)	yes (2)
Vascular crushing	no	no
Acquisition duration	4∶29 min	4∶33 min

*Labeling plane distance represents distance from the anterior commissure-posterior commissure (ACPC) line in the head-feet direction [20]. n.a. = not applicable.

### Post-processing: quantification

Matlab 7.12.0 (MathWorks, MA, USA) and Statistical Parametric Mapping (SPM) 8 (Wellcome Trust Center for Neuroimaging, University College London, UK) were used for post-processing and statistical analyses. For the Philips data, label and control pCASL images were pair-wise subtracted and averaged to obtain perfusion-weighted images. For the GE data, the perfusion-weighted images as directly provided by the scanner were used. Since the images as provided by GE did not incorporate motion correction, this was not applied to the Philips data. The perfusion-weighted maps of both vendors were quantified into CBF maps using a single compartment model [3], [17]:
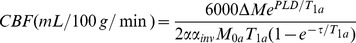
(1)where ΔM represents the difference images between control and label and M_0a_ the equilibrium magnetization of arterial blood. In Philips, ΔM was corrected for the transversal magnetization decay time (T_2_*) of arterial blood (48 ms) during the 17 ms echo time (TE) by e^TE/T2*^ [18]. PLD is the post-labeling delay (1.525 s), T_1a_ is the longitudinal relaxation time of arterial blood (1.650 s), α is the labeling efficiency (0.8), where α_inv_ corrects for the decrease in labeling efficiency due to the 5 and 2 background suppression pulses at GE (0.75) and Philips (0.83) respectively and τ represents the labeling duration (1.450 s and 1.650 s for GE and Philips respectively) [19]–[21]. The increase in label decay in the ascending acquired 2D slices in Philips-data was accounted for. GE has, but Philips has not, implemented a standard M_0_-acquisition where proton density maps are obtained with a saturation recovery acquisition using readout parameters identical to the ASL readout. These maps were converted to M_0a_ by the following equation:
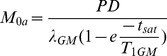
(2)where t_sat_ is the saturation recovery time (2 s), T_1GM_ is the relaxation time of gray matter (GM) tissue (1.2 s) and λ_GM_ is the GM brain-blood water partition coefficient (0.9 mL/g) [15], [22], [23]. For the Philips data, a single M_0a_-value was used for all subjects. This value was obtained in a previous study with the same center, scanner, head coil, pCASL protocol and a similar population (n = 16, 56% M, age 20–24 years) [24]. In short, cerebrospinal fluid T1 recovery curves were fitted on the control images of multiple time-point pCASL measurements, with the same readout, without background suppression. The acquired M_0_ was converted to M_0a_ by multiplication with the blood water partition coefficient (0.76) and the density of brain tissue (1.05 g/mL) [23], [25]. No difference was made between the quantification of GM and WM CBF.

### Post-processing: spatial normalization

A single 3D T1-weighted anatomical scan from each scanner for each subject (n = 44) was segmented into GM and white matter (WM) tissue probability maps. All CBF maps were transformed into anatomical space by a rigid-body registration on the GM tissue probability maps. The tissue probability maps were spatially normalized using the Diffeomorphic Anatomical Registration analysis using Exponentiated Lie algebra (DARTEL) algorithm, and the resulting normalization fields were applied to the CBF maps as well [26]. Finally, all normalized images were spatially smoothed using an 8×8×8 mm full-width-half-maximum Gaussian kernel, to minimize registration and interpolation errors.

### Data analysis

All intra-vendor reproducibility analyses were based on a comparison of session 1 with session 2 within each vendor (n = 22). All inter-vendor reproducibility analyses were based on a comparison of GE session 1 with Philips session 2, and GE session 2 with Philips session 1 (n = 44). In this way, the temporal physiological variation is expected to have an equal contribution to the intra- and inter-vendor reproducibility. All reproducibility analyses were based on the mean CBF of the two sessions, and on the mean and standard deviation of the paired inter-session CBF difference, denoted as ΔCBF and SD_ΔCBF_ respectively. The within-subject coefficient of variation (wsCV) - a normalized parameter of variation - was defined as the ratio of SD_ΔCBF_ to the mean CBF of both sessions:
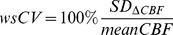
(3)


Reproducibility was assessed on a total GM and WM level, and on a voxel-level.

### Data analysis: total supratentorial GM and WM

Mean CBF-values of each session were obtained for the total supratentorial GM and WM. GM and WM masks were obtained by thresholding GM and WM probability maps at 70% and 95% tissue probabilities respectively. GM-WM CBF ratios were calculated individually. The significance of paired inter-session CBF differences (ΔCBF) was tested with a paired two-tailed Student’s t-test. The Levene’s test was used to test the significance of the difference between GE SD_ΔCBF_ and Philips SD_ΔCBF_, as well as between the inter-vendor SD_ΔCBF_ and both intra-vendor SD_ΔCBF_ [27]. Limits of agreement - defining the range in which 95% of future measurements is expected to lie - were defined as ΔCBF±1.96 SD_ΔCBF_ [28].

### Data analysis: voxel-level comparison

To assess spatial inter-vendor differences, CBF- and wsCV-values were computed for each voxel. For CBF, both sessions and all subjects were averaged. To test significant voxel-wise inter-vendor CBF differences, a Bonferroni-corrected paired two-tailed Student’s t-test was performed (using both sessions, n = 44). Individual histograms of CBF (25 bins, range 0–160 mL/100 g/min) were averaged to generate a group-level histogram. A wsCV histogram (25 bins, range 5–45%) was generated from the wsCV-maps. Both CBF and wsCV histograms were generated for the total supratentorial GM and WM of each vendor. Statistical significance was set to *p*<0.05 for all tests.

## Results

### Session timing

The number of days between intra-vendor sessions did not differ between vendors: 18.3±6.5 and 19.7±7.2 for GE and Philips respectively (independent sample Student’s t-test, *p* = 0.5). However, GE session 1 and session 2 took place earlier in the day compared to the Philips sessions (15 h26±4 h00 and 15 h55±3 h34 compared to 20 h16±2 h06 and 19 h47±2 h38 respectively, *p*<0.01).

### Total GM and WM

**Figure 2:**
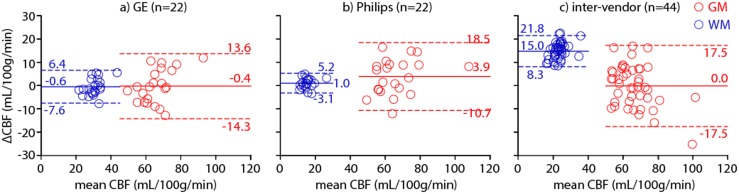
Bland-Altman plots. Intra-vendor a) GE (n = 22) and b) Philips (n = 22) and c) inter-vendor (n = 44) GM (red) and WM (blue) CBF differences are plotted against mean CBF. Continuous and broken lines indicate mean difference and limits of agreement (mean difference ±1.96 standard deviation of the paired difference) respectively. CBF = cerebral blood flow, GM = gray matter, WM = white matter.

The intra- and inter-vendor statistics are summarized in Table 2 and visualized by the Bland-Altman plots in Figure 2. GM CBF did not differ significantly between both vendors (*p* = 1.0), but WM CBF did (*p*<0.01). Likewise, the intra-vendor GM variances of the paired CBF differences did not differ between the two vendors whereas the WM variances did (*p* = 0.6 and *p* = 0.02 respectively). The GM-WM CBF ratios of both vendors differed significantly, the 2D readout (Philips) GM-WM ratio being approximately twice as large as the ratio of the 3D readout (GE) (*p*<0.01). Both the GM and WM intra-vendor wsCVs were similar to the inter-vendor wsCVs (Table 2), which is confirmed by the Levene’s test. The variance of GM inter-vendor CBF differences did not differ significantly from the variance of intra-vendor differences (*p* = 0.3 and *p* = 0.5 for GE and Philips respectively). For the WM, however, the variance of inter-vendor CBF differences did differ significantly from the Philips variance but not from the GE variance (*p* = 0.02 and *p* = 0.8 respectively).

**Table 2:** Inter-session statistics.

	GE	CI (n = 22)			Philips	CI (n = 22)			inter-vendor	CI (n = 44)		
GM mean CBF (mL/100g/min)	65.9	48.4	··	83.4	65.9	42.0	··	89.8	65.9	45.4	··	86.4
GM ΔCBF	−0.4	−3.5	··	2.8	3.9	0.6	··	7.2	0.0	−2.7	··	2.7
GM SD_ΔCBF_	7.1	4.8	··	9.4	7.5	5.1	··	9.8	8.9	7.0	··	10.9
GM lower LOA	−14.3	−18.2	··	−10.4	−10.7	−14.8	··	−6.6	−17.5	−20.9	··	−14.2
GM upper LOA	13.6	9.7	··	17.5	18.5	14.4	··	22.6	17.5	14.2	··	20.9
GM wsCV (%)	10.8	6.2	··	15.3	11.3	5.4	··	17.2	13.6	9.8	··	17.3
WM mean CBF (mL/100g/min)	30.5	22.0	··	39.0	15.4	9.1	··	21.7	22.9	15.6	··	30.3
WM ΔCBF	−0.6	−2.2	··	1.0	1.0	0.1	··	2.0	15.0	14.0	··	16.1
WM SD_ΔCBF_	3.6	2.4	··	4.7	2.1	1.4	··	2.8	3.5	2.7	··	4.2
WM lower LOA	−7.6	−9.6	··	−5.7	−3.1	−4.3	··	−2.0	8.3	7.0	··	9.6
WM upper LOA	6.4	4.4	··	8.3	5.2	4.1	··	6.4	21.8	20.5	··	23.1
WM wsCV (%)	11.7	9.5	··	13.9	13.8	12.2	··	15.4	15.0	13.7	··	16.4
GM-WM CBF ratio	2.2	1.9	··	2.5	4.3	3.4	··	5.2	2.9	2.4	··	3.4

Mean and ΔCBF represent the inter-session CBF mean and paired difference respectively.

The limits of agreement (LOA) represent ΔCBF±1.96 standard deviation of the paired difference (SD_ΔCBF_).

CI = confidence interval, CBF = cerebral blood flow, GE = General Electric, GM = gray matter,

WM = white matter, wsCV = within-subject coefficient of variation.

### Voxel-level comparison

**Figure 3:**
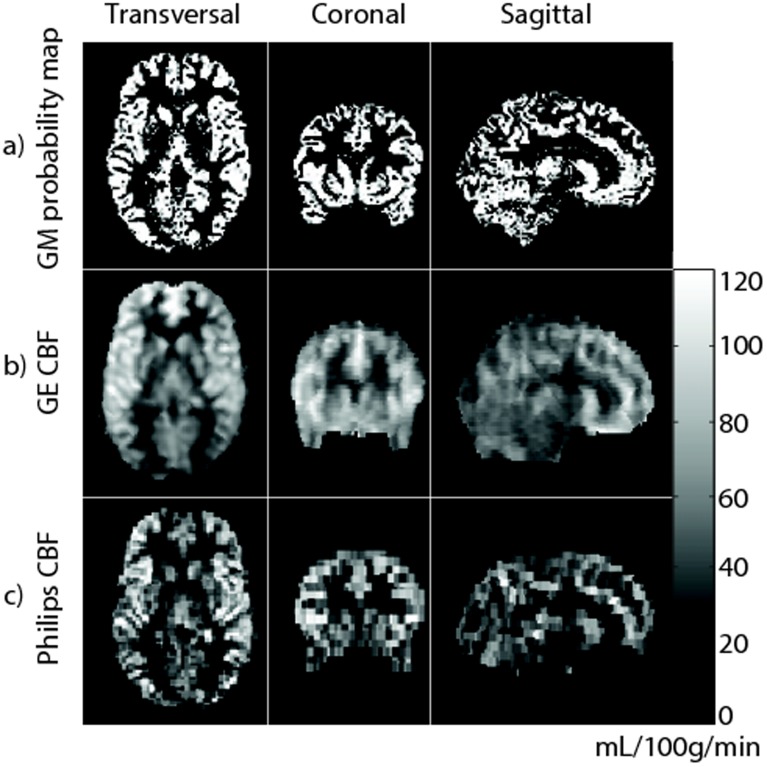
Cerebral blood flow maps of a representative subject of GE (b) and Philips (c), as compared to gray matter (GM) tissue probability map (a; for this example the GE 3D T1-weighted image was used). Maps are registered, re-sliced, skull-stripped and shown in native space.

**Figure 4:**
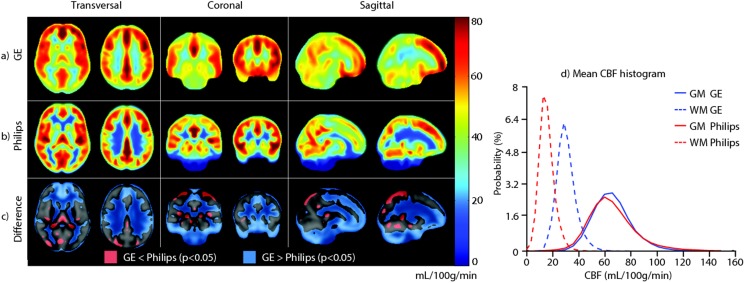
Mean cerebral blood flow (CBF) maps of all subjects (n = 22) are shown for GE (a) and Philips (b), averaged for both sessions. Voxel-wise significant inter-vendor differences are visualized by a binary parametric map projected on the gray matter (GM) probability map (c). Red voxels represent where GE <Philips, blue voxels represent where GE >Philips (Bonferroni corrected *p*<0.05). On the right, mean CBF histograms are shown for the total GM and white matter (WM) (d).

**Figure 5:**
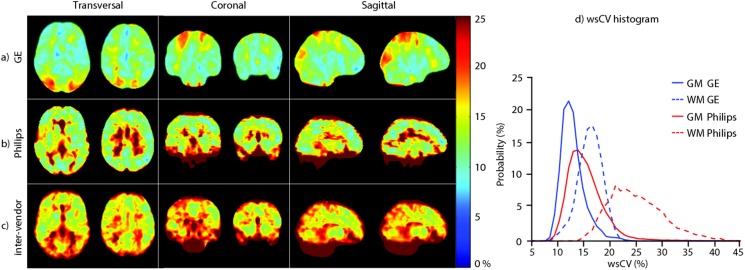
a) GE and b) Philips intra- and c) inter-vendor within-subject coefficient of variability (wsCV)-maps. d) wsCV histograms are shown on the right for the total gray matter (GM) and white matter (WM).

**Figure 6:**
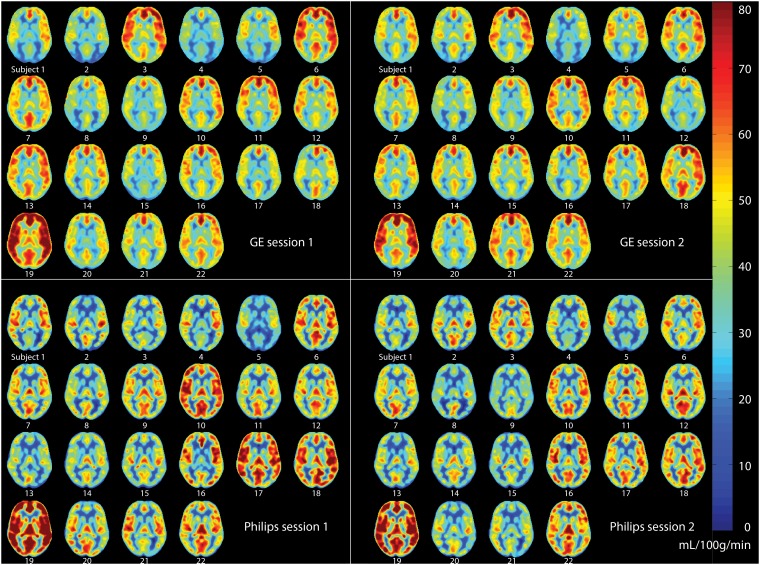
Single transversal cerebral blood flow slice of all subjects (n = 22) for GE (upper quadrants) and Philips (lower quadrants), session 1 (left quadrants) and session 2 (right quadrants), after spatial normalization.

Spatial CBF differences between GE and Philips are illustrated for a single subject and on group level in Figure 3 and 4 respectively. The spatial wsCV distribution is shown in Figure 5. In addition, Figure 6 provides an overview of spatial CBF differences between subjects, sessions and vendors for a single transversal slice. The main visual difference on all these maps was the homogeneity of GE compared to the heterogeneity of Philips, especially in the WM and in the z-direction. More specifically, the contrast between GM and WM was higher on the Philips CBF and wsCV-maps. Also within the GM, the CBF was more heterogeneous on the Philips maps compared to the GE maps. A CBF decrease and wsCV increase was observed in the posterior and superior regions on the GE maps and in the anterior-inferior and superior regions on the Philips maps. The GM CBF histograms were comparable between vendors (Figure 4d). The GE WM CBF histogram had a higher mean, but had the same shape as the Philips WM CBF histogram. The wsCV histograms, on the other hand, were less comparable (Figure 5d). The spatial GM wsCV distribution of Philips had a higher mean and was wider compared to GE. This difference in mean and spread was even larger for the WM.

## Discussion

The most important result of this study is that - despite several voxel-wise differences between vendors - there were no inter-vendor differences in mean CBF or wsCV on a total GM level. This can be explained by the fact that the variation between the sessions can for a large part be attributed to physiological factors, as was previously noted in single-vendor reproducibility studies [11], [29]–[31]. For clinical studies that focus on the GM in total, it may therefore be more important to minimize and account for physiological variation than to account for inter-vendor differences in ASL implementation.

A different picture arises for smaller GM regions or for the total WM. We observed several spatial differences between vendors which can mainly be explained by differences in the readout module. The most visually striking inter-vendor difference on all CBF- and wsCV-maps was in the WM. The GM-WM CBF ratio of the 2D readout (Philips) was twice as large as the ratio of the 3D readout (GE), which is in agreement with a previous readout comparison on a single Siemens scanner [13]. This can be explained by the larger extent of spatial smoothing of a spiral 3D readout (GE) compared to the 2D readout (Philips), which leads to more contamination of the GM signal into the WM and vice versa. Therefore, a 2D readout seems most suitable when the goal is to acquire uncontaminated GM or WM CBF – although the ability of ASL to measure WM CBF is debatable due to the long transit time of WM [32].

This difference in spatial smoothing may also explain the homogeneous GM appearance of the mean CBF and wsCV maps acquired with GE as compared to the more heterogeneous appearance of those acquired with Philips. In addition, it may explain the significant inter-vendor CBF difference within the subcortical GM since this area is surrounded by WM and therefore suffers more from smoothing with WM signal in GE (Figure 4c). Another explanation for the smaller spatial variation of GE, is its higher SNR compared to Philips. The SNR at GE is most probably higher because of the intrinsically high SNR of a 3D readout and because background suppression is more efficient for a single-volume readout as compared to a multi-slice readout [13]. In addition, parallel imaging was not available in the GE sequence, but was turned on in the Philips sequence. To what extent the heterogeneous appearance of the Philips CBF maps has a physiological origin or is rather the result of a too low SNR, cannot be differentiated with these data.

In regions with long arrival times - i.e. the posterior vascular territory and posterior watershed area - lower CBF and higher wsCV was observed in GE but not in Philips (Figures 4 and 5) [5]. This inter-vendor difference can be explained by differences in the effective post-labeling delay (PLD) between the readouts, even though both acquisitions had the same initial PLD (1525 ms). Whereas the 3D readout obtains all ASL signal for the total 3D volume at a single time-point - i.e. after 1525 ms PLD - the 2D readout obtains signal from each slice sequentially. With this multi-slice acquisition, each slice exhibits a longer effective PLD compared to its previous slice. This inferior-superior PLD increase of the 2D readout (Philips) allows the labeled blood more time to reach the superior slices compared to the homogeneous PLD of the 3D readout (GE). Therefore, the PLD may have been too short for the label to reach the superior slices in 3D (GE), whereas the effective PLD for the superior slices in 2D (Philips) was sufficient. These inter-vendor CBF differences and higher wsCV for GE in superior regions with long transit times are probably resolved by selecting a longer PLD for the 3D readout, such as 2000 ms [3].

Other prominent spatial inter-vendor CBF (Figure 4) and wsCV (Figure 5) differences were observed on the brain edges. We observed higher CBF and lower wsCV in anterior and inferior regions in Philips but not in GE. The prominent inferior CBF and wsCV differences (Figure 4c and Figure 5c) are partly due to the fact that these slices were simply not acquired by the 2D readout (Philips). With a 2D sequence, it is common practice to scan cerebral slices only as well as to optimize the PLD, T1 decay and background suppression for the cerebral slices. These issues do not apply for a 3D sequence, whose 3D slab usually has whole-brain coverage. The differences in the other areas can be explained by susceptibility artifacts from bone-air transitions at the paranasal sinuses and mastoid air cells present in the gradient-echo T2*-weighted readout implemented by Philips [33]. In addition, it is expected that the echo-planar imaging readout (Philips) exhibits geometric distortion in these regions [33]. The T2-weighted spin-echo readout employed by GE is much less sensitive to these artifacts, in comparison to the gradient-echo readout employed by Philips. For these reasons, a 3D readout is superior in regions such as the orbito-frontal lobe and cerebellum compared to a 2D readout. This especially favors the use of a 3D readout for clinical applications of ASL, since pathologies in these regions could remain undetected on a 2D readout [34]–[36].

A limitation of the current study is that we did not acquire spatial M_0_-maps with the same readout in Philips. By employing a voxel-wise normalization of the ASL-signal, these maps would have opposed the T_2_^*^ susceptibility effects, since these will be approximately equally large for the ΔM and M_0_-map. Therefore, Philips spatial M_0a_-maps could have improved quantification in regions of air-tissue transitions, which may have diminished the inter-vendor variation to a certain extent. However, the added value of spatial M_0_-maps is limited since they cannot improve the lower SNR of the gradient-echo readout (Philips) near the air-tissue transitions. Therefore, the inter-vendor reproducibility in these regions is expected to remain low.

The current study may also be limited by the inter-vendor calibration of quantification parameters. These may remain arbitrary, mostly because they have been derived from simulations rather than measurements. One example is the inter-vendor differences in labeling efficiency due to a different number of background suppression pulses (5 and 2 for GE and Philips respectively) [21]. One way to deal with this is to scale to a phase-contrast MRI sequence of the main feeding arteries [20]. However, this would shift the inter-vendor CBF variation from the ASL-sequence towards the phase-contrast MRI measurements.

Inter-vendor CBF and wsCV differences were observed on a voxel-level but not on the total GM level. Apparently, the effects of the abovementioned readout differences do cancel out when sufficient GM voxels are averaged. There are several explanations for this observation. First, the higher SNR of the 3D module may be important on a voxel-level, but if sufficient GM voxels are averaged physiological variation seems to outnumber the SNR differences between the readout modules. Second, the smoothing of the GE 3D readout averages signal from multiple GM voxels which increases SNR and subsequently decreases the wsCV within a single voxel. This effect is similar to averaging signal from multiple GM voxels of the 2D readout in post-processing. Therefore, this difference of spatial signal averaging between both readouts becomes apparent on a voxel-level but is negligible when all GM voxels are averaged.

It should be acknowledged that this study evaluated healthy controls only. The abovementioned inter-vendor readout differences could become more or less important in patients, considering the different spatial CBF variation in patients compared to healthy controls. Furthermore, these inter-vendor differences should not be generalized to all MRI vendors. Visual readout differences between GE and Siemens, who both use a 3D approach, may be smaller than the readout differences in the current study [13].

In conclusion, the current study shows that pCASL results do not differ between vendors on a total GM level. Therefore, the reliability of averaged CBF-values for the total GM can be expected to be equal in single- and multi-vendor studies. However, the reliability of measurements in GM regions or in the WM, is impeded by differences between the readout modules of both vendors. Therefore, our results strongly encourage the standardization of ASL implementations among vendors, which was also advocated by the recent ASL consensus paper [3].
